# Determination of Extravasation Effects of Nal-Iri and Trabectedin and Evaluation of Treatment Options for Trabectedin Extravasation in a Preclinical Animal Model

**DOI:** 10.3389/fphar.2022.875695

**Published:** 2022-06-03

**Authors:** Omar Keritam, Viktoria Juhasz, Christian Schöfer, Christiane Thallinger, Marie-Bernadette Aretin, Gernot Schabbauer, Johannes Breuss, Matthias Unseld, Pavel Uhrin

**Affiliations:** ^1^ Institute of Vascular Biology and Thrombosis Research, Center for Physiology and Pharmacology, Medical University of Vienna, Vienna, Austria; ^2^ Department for Cell and Developmental Biology, Center for Anatomy and Cell Biology, Medical University of Vienna, Vienna, Austria; ^3^ Clinical Division of Infectious Disease, Department of Internal Medicine I, Medical University of Vienna, Vienna, Austria; ^4^ Pharmacy Department, General Hospital Vienna, Vienna, Austria; ^5^ Clinical Division of Palliative Care, Department of Internal Medicine I, Medical University of Vienna, Vienna, Austria

**Keywords:** extravasation, nanoliposomal irinotecan, trabectedin, hyaluronidase, DMSO, tacrolimus

## Abstract

**Background:** Extravasation during chemotherapy administration can lead to dangerous adverse effects ranging from pain to tissue necrosis. Evidence-based data about prevention and treatment of extravasation injuries of some clinically used compounds still remains elusive. This work aimed to investigate, in a preclinical mouse model, the effects of extravasation of two chemotherapeutic agents, nanoliposomal irinotecan (nal-Iri) and trabectedin. In addition, we aimed to study treatment options for injuries induced by extravasation of these substances.

**Methods:** Mice were subcutaneously injected with nal-Iri or trabectedin applied in clinically used concentration. Doxorubicin was used as a positive control. In subsequently performed experiments, hyaluronidase, DMSO and tacrolimus were tested as potential treatments against extravasation-induced injuries by trabectedin. Systemic effects were analyzed by observation and documentation of the health status of mice and local reactions were measured and graded. In addition, hematoxylin-eosin stained histological sections of the treated skin areas were analyzed.

**Results:** Of the two tested substances, only trabectedin showed vesicant effects. Subcutaneous injection of trabectedin caused erythema formation in mice by day two that was progressing to skin ulcerations by day five. Furthermore, we found that topical treatment of mice with tacrolimus or DMSO reduced the vesicant effects of trabectedin. The results observed *in vivo* were supported microscopically by the analysis of histological sections.

**Conclusions:** We recommend classifying trabectedin as a vesicant agent and nal-Iri as a non-vesicant agent. Furthermore, our results obtained in a preclinical model suggest that tacrolimus and DMSO might be suitable treatment options of trabectedin extravasations, a finding that might be further utilized in clinical studies.

## Introduction

The term “extravasation” describes the escape of drugs, injected into blood vessels, into the surrounding tissue. In case of extravasation during cytostatic or cytotoxic therapy, serious tissue damage can occur, which can progress to tissue necrosis. Such extravasation can therefore—especially in the field of oncology—present an emergency with an acute need for action. The incidence of symptomatic extravasation varies between 0.5% and 6% (Barlock et al., 1979; Kassner, 2000; Langstein et al., 2002; Goolsby and Lombardo, 2006).

Different risk factors for extravasation are to be considered. They can be summed up into four categories: patient-specific (e.g., cardiovascular disease, obesity), health care-specific (e.g., time pressure, establishment of intravenous access), procedure-specific (e.g., volume and duration of infusion, quality of equipment) and substance-specific (e.g., chemical properties, time of exposure) (Larson, 1982; Boschi and Rostagno, 2012; Pérez Fidalgo et al., 2012; de Wit et al., 2013; Reynolds et al., 2014). Timely identification of these potential risk factors is crucial to successfully prevent or minimize the risk for extravasation injuries (Goolsby and Lombardo, 2006; Schulmeister, 2011).

Possible early symptoms of extravasation injuries can include pruritus, paraesthesia, burning pain, induration, edema, erythema, epidermolysis, or blistering. Late symptoms can be ulcerations or necrosis with chronic pain symptoms. As an example, the analysis of the incidence of clinical symptoms in a cohort of 545 patients that had been treated with chemotherapeutic drugs from January 1994 to December 2015, showed that 18% of patients with chemotherapy drug extravasation had clinical symptoms without visible lesions, 73% developed cutaneous superficial lesions, and 9% ulcerated lesions (Onesti et al., 2017). The clinical course of tissue damage induced by a cytostatic agent depends very strongly on the toxicity of the used substance. In the context of extravasation, chemotherapeutic agents are divided into three categories: vesicants, irritants and non-vesicants (Ener et al., 2004). Vesicant substances can have serious tissue-damaging effects, which can range from diffuse tissue damage to blistering, epidermolysis, ulceration, or necrosis. Vesicants can be divided into two groups: DNA binders and DNA non-binders. Adverse effects of DNA binders are usually more dangerous than those caused by DNA non-binders, as binding to the DNA allows these substances to persist over a prolonged time in the tissue. It is assumed, that DNA binders, after their release from necrotic cells might be endocytosed by healthy cells around the extravasation site. This way lesions become larger, deeper, and more painful, and the extravasation damage may become chronic (Ener et al., 2004). DNA non-binders are metabolized and neutralized quicker and this way they may cause only mild- to moderate lesions that heal in a significantly shorter time (Schulmeister, 2011; Onesti et al., 2017). Irritant substances may cause local pain, with or without inflammation and, e.g., through irritation of the venous vessels may cause vasospasm leading to a venous flow obstruction, which may raise the risk of extravasation due to the resulting increased hydrostatic pressure. Chemically-induced phlebitis is often associated with this mechanism (Onesti et al., 2017). Non-vesicants, on the contrary, cause no local or systemic damage (Ener et al., 2004).

Treatment options depend on the stage of extravasation and the type of the administrated substance. Intervention should be carried out as early as possible to prevent comorbidities and delays in primary therapy (Scuderi and Onesti, 1994; Shenaq et al., 1996). Treatment protocols proposed in the literature vary widely ranging from conservative to aggressive methods (Brown et al., 1979; Zenk et al., 1981; Siwy and Sadove, 1987; Falcone et al., 1989; Bertelli, 1995; Friedman, 1998; Harris et al., 2001; Wilkins and Emmerson, 2004). Unfortunately, data on antidotes that can be used to neutralize effects of a specific vesicant are available only in a limited number of cases.

In our study we aimed to investigate the damaging effects of the nanoliposomal form of the topoisomerase-inhibitor irinotecan (nal-Iri) and of the tetrahydroisoquinoline alkaloid trabectedin. Nal-Iri is used for treatment of refractory pancreatic cancer (Bien et al., 2016) and trabectedin is therapeutically utilized in patients with soft tissue sarcomas and ovarian tumours (Brodowicz, 2014; Ventriglia et al., 2018). While irinotecan has been classified as a non-vesicant (Jordan et al., 2005), trabectedin exhibited severe adverse effects in human oncological patients, as shown in case reports (Theman et al., 2009; Yoshimi et al., 2017). Yet, the adverse effects of extravasation of neither of these substances have been directly assessed in a preclinical mouse model. After characterising effects of these compounds, we aimed to study potential treatment options using hyaluronidase injection or topical treatment with DMSO or tacrolimus ointment (Bertelli et al., 1994; Bertelli, 1995; Ohtsuki et al., 2018) to prevent or minimize injuries induced by extravasation of these chemotherapeutics.

## Materials and Methods

### Animals

BALB/c mice derived from Division of Laboratory Animal Science and Genetics (Himberg, Austria) were bred at the Medical University of Vienna under specific pathogen-free conditions. For the experiments, mice aged eight to 12 weeks were used. All experimental procedures of this study were carried out under the approval of the Animal Experimental Ethics Committee of the Medical University of Vienna and the Austrian Federal Ministry for Education, Science and Research (Permission Nr. BMBWF-66.009/0196-V/3b/2019).

### Subcutaneous treatment of mice with trabectedin, nal-Iri and doxorubicin within a 2 day period

Trabectedin (Yondelis®) was obtained from Pharma Mar, S.A. Madrid, Spain, nal-Iri (Nal-Irinotecan, Onivyde®) from Les Laboratoires Servier Industrie, Gidy, France) and doxorubicin (Adriblastin®), used as a positive control, from Pfizer Corporation Austria Ges.m.b.H., Vienna. The quantities of the used substances were determined in a preliminary test in which we intended to inject them subcutaneously in ascending order (150 µl > 200 µl > 250 µl > 300 µl) in order to determine which of these amounts would suffice to cause a visible effect. We applied these substances in concentrations used for treatment of human patients, as specified in result section. As a negative control, to eliminate any bias caused by mechanically triggered effects, we administered 0.9% NaCl.

After removing the hair in the vicinity of the scapula (clean shave), the above substances were subcutaneously injected with plastic syringes and 30-gauge needles. During the observation period, treated mice were kept in separate cages, and tramadol (10–20 mg/kg) was added to the drinking water to relieve the animals from pain.

In the pilot experiment, the observation period was 48 h. The health status of the animals was continuously assessed, both with regard to their general condition and with regard to local reactions. For documentation purposes, the animals were photographed daily under anaesthesia, i.e., at the time of treatment, 24 h after treatment and at the end of the experiment when they were sacrificed by cervical dislocation.

### Investigation of Antidotes Against Extravasation Injuries of Trabectedin

Following the examination of adverse effects caused by nal-Iri or trabectedin within a 2 day period, we aimed to determine possible therapeutic interventions against trabectedin induced injuries. To this end, hyaluronidase (Hylase®, Dessau, Riemser Pharma GmbH, Berlin), 0.1% tacrolimus monohydrate ointment (Protopic®, Leo Pharma A/S, Ballerup, Denmark) and dimethyl sulfoxide (DMSO, Cat. Nr. 76855, Sigma Aldrich, Saint Louis, MO, United States), were tested as possible antidotes in an experiment lasting 5 days. To this end, animals were divided into positive control group (trabectedin), hyaluronidase group (hyaluronidase treatment after trabectedin injection), tacrolimus group (tacrolimus ointment treatment after trabectedin injection) and DMSO group (DMSO treatment after trabectedin administration).

Briefly, animals were injected with trabectedin as described in the previous section and in addition, hyaluronidase, tacrolimus or DMSO were administered subsequently. We injected overall 20 µl of hyaluronidase (150 IU/ml) subcutaneously, with a Hamilton syringe, around the injection site of trabectedin, immediately after the administration of trabectedin. Tacrolimus ointment and DMSO were applied topically at the site of trabectedin injection three times daily until the end of day five. The experimental animals were monitored/treated for 5 days, and the injection site was macroscopically inspected and photographed daily.

### *In Vivo* Assessment

Assessment schemes for the severity of the local and systemic reactions were derived from the grading systems of the “Common Terminology Criteria for Adverse Events” (National Cancer Institute, N. I. o. H., U.S. Department of Health and Human Services, 2010), the “Infusion Nurse Society” (Infusion Nursing Standards of Practice, 2006) and based works of Amjad et al. (2011) and Alexander (2020). Skin damage was graded using the following scheme: 0° = no damage, 1° = skin pallor, 2° = erythema, 3° = ulcer/necrosis (assigned when the area of ulceration/necrosis comprised at least 15% of the total afflicted area). The area of the affected skin was measured.

### Processing of Skin Samples and Staining

After cervical dislocation, skin samples around the injection sites were cut out and histological sections were prepared from areas of the greatest macroscopically visible effects. Briefly, samples were kept in 4% paraformaldehyde (PFA) solution at 4°C overnight and transferred into 0.1% PFA solution the next day. They were embedded in paraffin using the TBS88 Paraffin Embedding System (Medite Medical GmbH, Burgdorf, Germany) and after storing at +4°C used for preparing series of sections with thickness of 5 µM using the Leica microtome (Wetzlar, Germany).

Before hematoxylin/eosin (H&E) staining, sections were deparaffinized with xylene for 1 hour. The Continuous Linear Stainer COT20 (Medite) was then used for the H&E staining. Briefly, a sequence of following steps, each taking two and a half minutes, was executed: Histolab-Clear, ethanol (100%–70%), H_2_O distilled, hematoxylin (hematoxylin acidic according to MAYER), flowing water (×5), eosin, running water, ethanol (70%–100%), Histolab-Clear and xylene exposure.

### Microscopy

The Olympus BX61VS slide scanner (Olympus, Tokyo, Japan) was used to image the stained histological H&E sections using ×10 objective lens and analyzed using the Olympus OlyVIA Ver.2.9.1 software.

### Statistics

IBM SPSS Statistics 27.0 and GraphPad Prism 5 software were used for statistical analysis. Nominal data were analyzed using χ2 test, ordinal data by Kruskal-Wallis analysis of variance and metric data by simple analysis of variance (ANOVA, ANalysis Of VAriance) and subsequent post-hoc tests with a Tukey-B correction. A bilateral significance level of 5% and a statistical power of 90% were applied. Graphs were created using GraphPad Prism 5 software.

## Results

### Extravasated Trabectedin but not Nanoliposomal Irinotecan Caused Local Adverse Effects Within a 2-Day Treatment

To assess the extent of skin damage caused by the tested chemotherapeutics nal-Iri and trabectedin and by the positive control substance doxorubicin, we used four mice in each group. We observed these mice several times daily and recorded images of the injected skin area at 24 and 48 h when the animals were sacrificed and the skin around the injection site was excised and used for histological assessment.

**FIGURE 1:**
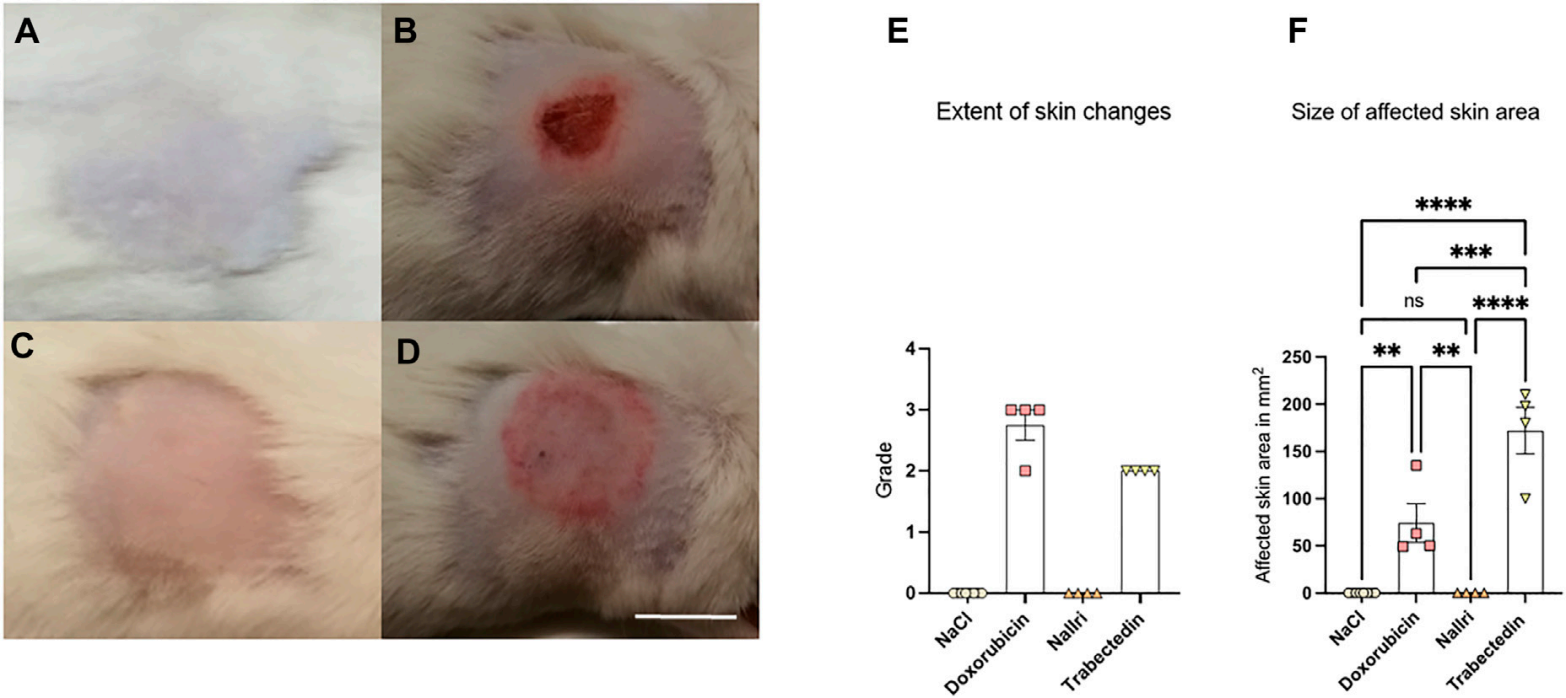
Representative images of treated area of mice at day two after subcutaneous injection of **(A)** 150 µl or 300 µl NaCl (grade 0), **(B)** 150 µl doxorubicin (grade 3), **(C)** 300 µl nal-Iri (grade 0), **(D)** 150 µl trabectedin (grade 2). **(E)** Grading of skin changes where grade 0 was assigned to all mice treated with NaCl or nal-Iri. **(F)** Size of affected skin area with at least grade 2 statistically analyzed using ANOVA followed by a post-hoc test with a Tukey-B correction. **p < 0.01, ***p < 0.005, ****p < 0.0001, ns = not significant. Scale bar = 1 cm.

Originally, we intended to inject increasing amounts of these compounds by applying 150 μl, 200 μl, 250 μl and 300 µl in ascending order. However, subcutaneous injection of doxorubicin (applied at 2 mg/ml) and of trabectedin (applied at 0.05 mg/ml) induced already at the lowest volume of 150 μl substantial skin changes (Figures 1B,D). In case of nal-Iri (applied at 4.3 mg/ml concentration, Figure 1C), and 0.9% NaCl (used as a negative control where we injected to four mice in each group either 150 µl or 300 μl, respectively, Figure 1A), neither significant changes in the skin color nor presence of ulceration/edemas in the skin of such treated animals were found even at the highest applied volume of 300 µl. Therefore, in case of all mice treated with nal-Iri or NaCl, their lesions were classified with grade 0 (Figure 1E). The average area of the skin discoloration in both these experimental groups was assigned to 0 ± 0 mm^2^ (Figure 1F).

In case of doxorubicin treatment, one mouse developed erythema without ulceration (classified as grade 2) and three mice developed skin ulceration (classified as grade 3, Figure 1E). In case of trabectedin, all four treated animals developed erythema and edema (Figure 1E). The average area of the skin discoloration in doxorubicin group was 74 ± 41 mm^2^ and in trabectedin group 172 ± 50 mm^2^ (Figure 1F).

Treatment of mice with any of the above substances (doxorubicin, trabectedin or nal-Iri) did not cause an impairment in the general health- or nutritional status or their appearance. Furthermore, no obvious changes in digestion or abnormal behavior were observed in any of treated animals.

**FIGURE 2:**
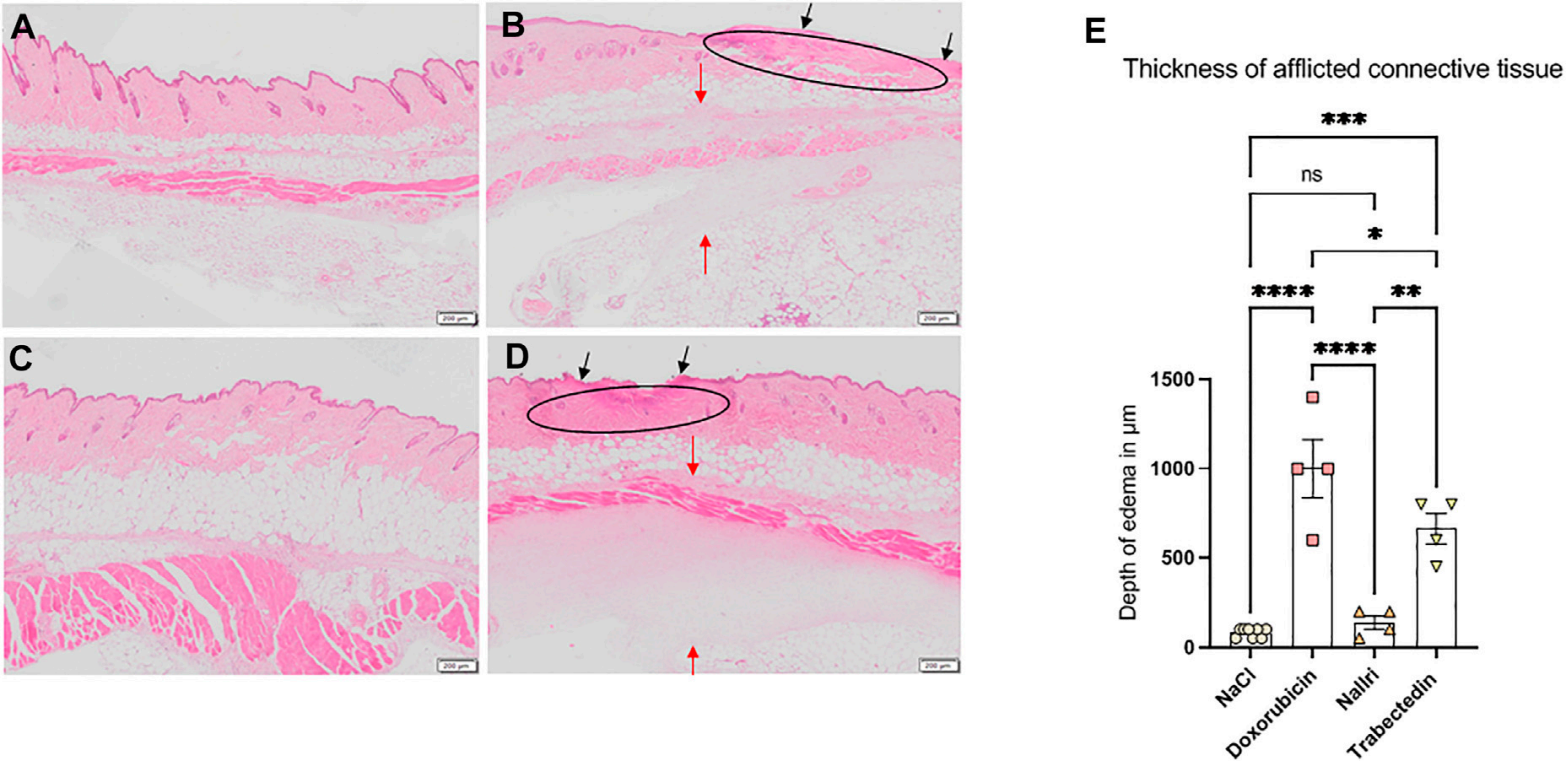
Images of H&E stained sections of affected skin area at day two of treatment with **(A)** 300 µl NaCl, **(B)** 150 µl doxorubicin, **(C)** 300 µl nal-Iri, **(D)** 150 µl trabectedin. Histological sections in B and D show the loss of epidermal continuity (black arrows), replacement of the dermis with granulation tissue (ellipse) more pronounced in B than in **(D)**. Due to edematous changes, the loosening of the connective tissue occurs (red arrows in B and D). **(E)** Thickening of afflicted connective tissue layer statistically analyzed using ANOVA followed by a post-hoc test with a Tukey-B correction. *p < 0.05, **p < 0.01, ***p < 0.005, ****p < 0.0001, ns = not significant.

We further assessed morphological changes in the skin around the injection site by H&E staining. For detailed assessment of sections, we took into consideration different factors including dermal remodeling with granulation, loss of epidermal continuity, thickening of epidermis, presence of loosened connective tissue and the size of the edema (maximal thickness observed in tissue sections). The most typical phenotypic skin changes from each group of animals are presented on Figures 2A–D. The analysis of histological sections revealed that three of four of doxorubicin-treated mice and one of four of the trabectedin-treated mice showed dermal remodeling with granulation and a loss of epidermal continuity (Figures 2B,D, respectively), while the mice of the nal-Iri and NaCl groups did not show such changes (Figures 2A,C, respectively). Furthermore, three of four of doxorubicin-treated mice and two of four of trabectedin-treated mice (Figures 2B,D, respectively), but none of the mice in the NaCl- or nal-Iri group (Figures 2A,C, respectively) showed a segmentally thickened epidermal layer.

We found that the thickness of the afflicted tissue possibly reflecting the degree of edema formation was 1000 ± 327 µM in doxorubicin group, 663 ± 170 µM in trabectedin group, 138 ± 75 µM in nal-Iri group and 88 ± 25 µM in NaCl control group (Figure 2E). Statistical analysis using ANOVA followed by a post-hoc test with a Tukey-B correction revealed no significant difference between the NaCl groups and the nal-Iri group, but a significant difference between the two mentioned groups and the trabectedin and doxorubicin groups (Figure 2E).

Altogether, the data obtained within this 2-day experiment showed the non-vesicant properties of nal-Iri and revealed significant vesicant properties of trabectedin. Furthermore, these experiments confirmed the widely-known vesicant-properties of doxorubicin (Barlock et al., 1979; Kassner, 2000).

### DMSO and Tacrolimus Reduced Adverse Effects of Trabectedin Extravasation

Following the investigation of the adverse effects caused by nal-Iri and trabectedin, we aimed to determine possible therapeutic interventions that might lead to minimizing adverse effects of trabectedin extravasation. To this end, we injected trabectedin subcutaneously and such treatment was followed by subcutaneous injection of hyaluronidase around the injection site of trabectedin or by topical application - three times daily around the injection site - of tacrolimus ointment or DMSO. Trabectedin group was comprised of seven mice, hyaluronidase group of six mice, tacrolimus group of seven mice and DMSO group of six mice. We followed the mice daily for a period of 5 days.

**FIGURE 3:**
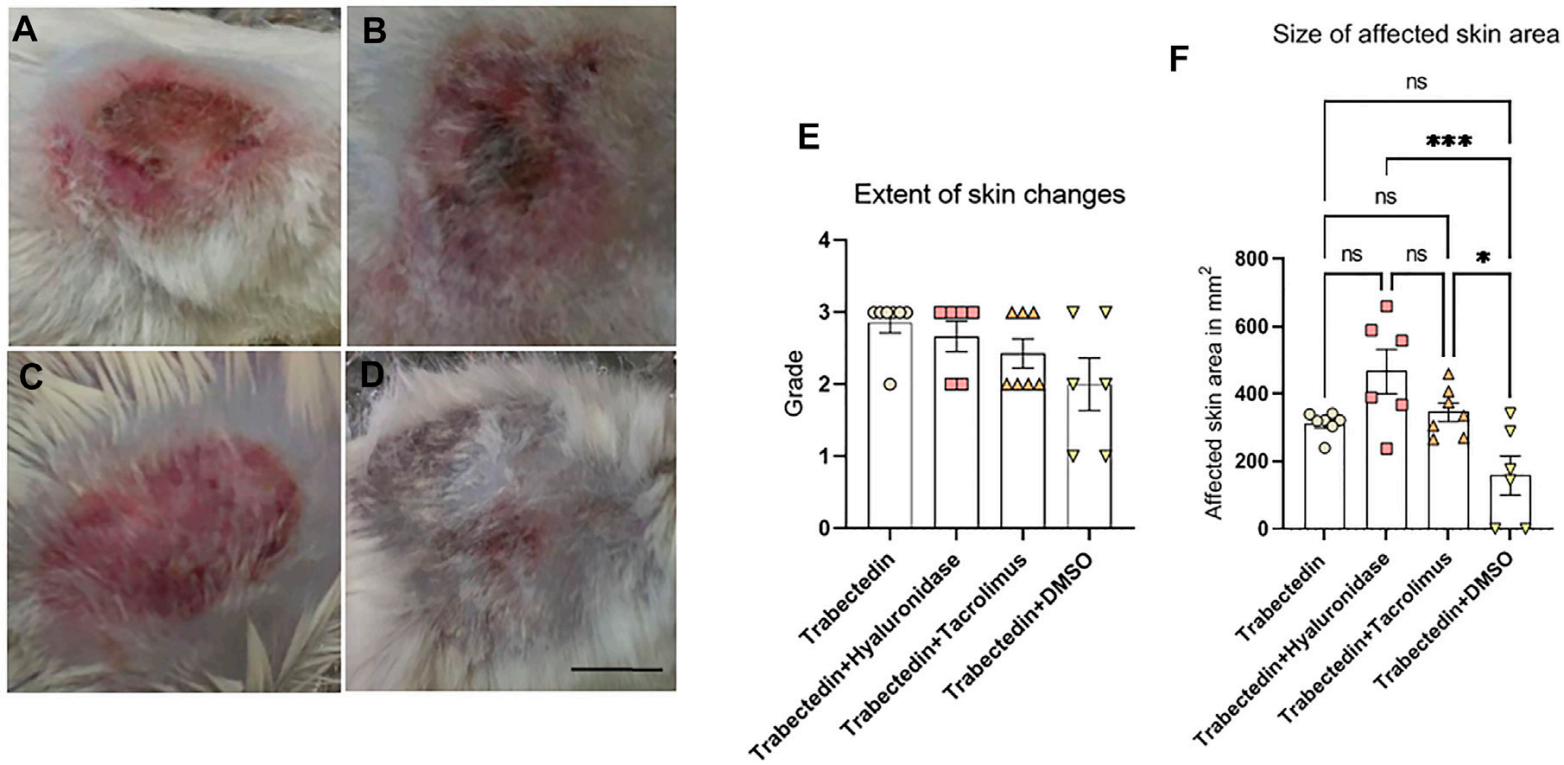
Representative images of treated area in mice at day five after treatment with **(A)** trabectedin (grade 3), **(B)** trabectedin and hyaluronidase (grade 3), **(C)** trabectedin and tacrolimus (grade 2), **(D)** trabectedin and DMSO (grade 2). **(E)** Extent of skin changes assessed by grading. **(F)** Size of affected skin area in lesions graded at least as grade 2, statistically analyzed using ANOVA followed by a post-hoc test with a Tukey-B correction *p < 0.05, ***p < 0.005, ns = not significant. Scale bar is 1 cm.

Among these animals, two of six mice treated with DMSO started to show signs of lethargy on day five, otherwise no impairment of activity or any changes in the nutritional status or digestion were found. Representative figures of the skin area around the injection site observed at day five of mice injected with 150 µl of 0.05 mg/ml trabectedin only, or with additional subsequent treatment with hyaluronidase, tacrolimus ointment or DMSO are shown on Figures 3A–D.

The assessment of the macroscopically discernable skin changes at day five revealed that six of seven mice of trabectedin group developed ulceration and necrosis (grade 3) while one mouse exposed to trabectedin developed only erythema and edema classified as grade 2 (Figure 3E). In the hyaluronidase group, grade 3 was observed in five of six experimental mice and the affected skin area of one mouse was characterized as grade 2 (this difference in the extent of macroscopically observed pathological skin changes compared to the positive control as analyzed by Kruskal-Wallis analysis of variance, was not statistically significant, *p* = 0.435). The pathological skin changes in mice treated with trabectedin and tacrolimus were less pronounced compared to the control trabectedin group (*p* = 0.037), as only three of seven mice developed grading three. Significant improvement in comparison to the control trabectedin group was also seen in the trabectedin/DMSO group (*p* = 0.017), as only two of six mice exhibited signs of ulcerations at day five (Figure 3E).

Analysis of the size differences of affected areas (Figure 3F) revealed that macroscopically visible damages were significantly more spread out in the hyaluronidase group (467 ± 160 mm^2^) than in the trabectedin control group (313 ± 35 mm^2^). In case of mice exposed to trabectedin and treated for 5 days with tacrolimus, the affected area (346 ± 73 mm^2^) did not significantly differ from the control group. In contrast, a 5-day treatment with DMSO resulted in a smaller affected area (159 ± 143 mm^2^), yet this difference was not statistically significant. Altogether, these findings on the size of the affected area concur with the above presented macroscopic classification of pathological skin changes in the control and tested experimental groups.

**FIGURE 4:**
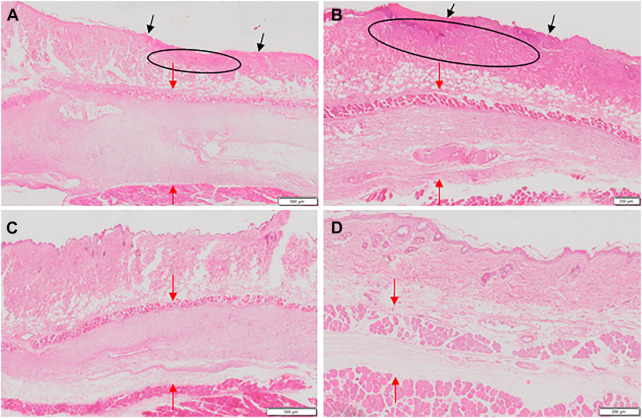
Images of H&E stained sections of affected skin area at day five of treatment with **(A)** trabectedin, **(B)** trabectedin and hyaluronidase, **(C)** trabectedin and tacrolimus, **(D)** trabectedin and DMSO. Histological sections in A and B show a loss of epidermal continuity (black arrows in A and B) and a replacement of the physiologic structure of the dermis by the granulation tissue (ellipse), more pronounced in B than in A. Due to the edematous changes, the connective tissue was severely loosened (red arrows) in A, B, C and D.

Next we evaluated obtained histological skin sections and found that the microscopic appearance corresponded to macroscopic findings presented above. Representative images of the H&E stained histological sections of the skin areas around the injection sites are shown in Figures 4A–D.

In the positive control group (trabectedin), sections of all seven mice showed dermal remodeling/granulation as well as loosened connective tissue and loss of epidermal continuity. Thickening of the epidermis was found in samples of five mice of this group. In the hyaluronidase group consisting of six mice, sections of five mice exhibited dermal remodeling/granulation and loosened connective tissue while all six mice had thickened epidermis. Overall, the differences in these parameters in the hyaluronidase group in comparison to the positive control group, as determined using χ2 test, were not statistically significant. In the tacrolimus group, no dermal remodeling/granulation was found in any of the analyzed samples (*p* < 0.001) and no loss of epidermal continuity was observable in samples from five of seven of these mice (*p* = 0.005). A better outcome, as assessed by the above parameters, was observed also in the DMSO group where only two of six mice showed dermal remodeling/granulation and thickening of epidermis (*p* = 0.009). In contrast, loosened connective tissue and loss of epidermal continuity was found in four of six mice from this group (*p* > 0.05).

We also quantified edema formation by assessing the thickness of the affected tissue layer. In the positive control group edema thickness was 817 ± 349 μM, in the hyaluronidase group 633 ± 281 μM, in the tacrolimus group 650 ± 152 µM and finally, 517 ± 343 µM in the DMSO group. Due to insufficient integrity of the edema region in the respective paraffin tissue samples, this evaluation did not include the assessment of one mouse of the trabectedin group and one mouse of the tacrolimus group. Overall, the differences between the test groups and the positive control trabectedin group were not statistically significant.

## Discussion

A clinical investigation of the effects of the extravasation of chemotherapeutic agents in the context of a prospective clinical study is often associated with many hurdles and obstacles and might not be easily ethically justifiable. To avoid this problem, in our study of local and systemic effects of extravasation of the oncological therapeutics nanoliposomal irinotecan (nal-Iri) and trabectedin, we used a preclinical mouse model. To the best of our knowledge, neither the effects of nal-Iri nor of trabectedin extravasation have been systematically tested in a preclinical mouse model.

Briefly, subcutaneous injection of nal-Iri did not cause any systemic reaction in the observed period, that is, no pathological changes in the general condition, nutritional condition or in behaviour were observed. Furthermore, such injection did not result in any harmful effects on the skin, as the appearance of the treated skin areas of these mice in the nal-Iri group was indistinguishable from that of the mice in the NaCl groups. Overall, neither macroscopic nor histological differences regarding the local reaction between the two groups could be detected. In summary, these results agree with the previous literature, according to which damage induced by nal-Iri was, due to the non-vesicant nature of irinotecan (Jordan et al., 2005), not to be expected, and since no adverse effects of extravasation of its nanoliposomal form administrated to metastatic pancreatic cancer patients were reported (Wang-Gillam et al., 2016).

After demonstrating vesicant effects of extravasated trabectedin in a preliminary experiment lasting for 2 days, we aimed to investigate possible treatment options. We treated mice that have been challenged with trabectedin injection with either hyaluronidase, tacrolimus or DMSO within a 5 day period.

Hyaluronidase, an enzyme that temporarily disintegrates tissue due to the degradation of hyaluronic acid, has been in earlier studies suggested as antidote for treating of nafcillin extravasation (Zenk et al., 1981) and later on for treating of plant vinca alkaloids extravasation (Bertelli et al., 1994; Jordan et al., 2005; Goolsby and Lombardo, 2006; Reynolds et al., 2014). Yet, in our study, the use of hyaluronidase did not result in successful prevention or minimization of extravasation injuries. To the contrary, its use tended to strengthen the adverse effects of extravasated trabectedin. The area of macroscopically visible changes reaching at least grade 2 at day five of treatment, in fact, was the largest among all four experimental groups. Histological parameters were comparable to the control trabectedin group.

Tacrolimus is a substance with anti-inflammatory- and immunosuppressive properties used for treatment of patients after organ transplantation (Plosker and Foster, 2000; Bush and Lin, 2006) and applied topically as an ointment for treatment of atopic dermatitis patients (Ohtsuki et al., 2018). In our study, topical application of tacrolimus ointment attenuated the extent of macroscopic skin damage, as revealed by grading of the observed changes at day 5 of the treatment, as only three of seven mice developed grade 3. Although the affected area of macroscopically visible changes in mice exposed for 5 days to tacrolimus did not significantly differ from the control trabectedin group, histological findings revealed that the use of tacrolimus decreased dermal remodeling/tissue granulation and reduced the loss of epidermal continuity in comparison to the positive control group.

Topical application of dimethylsulfoxide (DMSO) has been recommended for treating of anthracyclines-, mitomycin C- or cis-platin extravasation induced tissue necrosis in clinical studies, probably due to its effect as a radical scavenger, anti-inflammatory and vasodilatory activity (Bertelli, 1995; Rosenstein, 1999; Jordan et al., 2005; de Wit et al., 2013). The above mentioned drugs, similarly as trabectedin, possess DNA-binding properties and therefore persist longer in the tissue, promoting tissue damage and necrosis (Zewail-Foote and Hurley, 1999; Ener et al., 2004; de Wit et al., 2013). Mechanistically, DMSO penetrating deeply into the tissue might foster dilution of these substances (Olver et al., 1988; Bertelli, 1995) and consequently counteract these adverse effects. Use of DMSO in our study diminished formation of ulceration/necrosis around the injection site as only two of six mice exhibited signs of ulcerations at day five, two mice presented grade 2 and two mice developed grade 1. In addition, suppression of dermal remodeling/granulation was seen in mice of the DMSO-treated group. However, two of the six mice presented signs of apathy on day five of the treatment and had to be sacrificed shortly thereafter. Although we did not investigate the cause of a sudden worsening of their health status, we think that it might be linked to a release of the trabectedin into systemic circulation.

In summary, the local effects observed in our study suggest that between the two tested substances nal-Iri and trabectedin, only trabectedin has the damage potential of a vesicant. Altogether, obtained data does not support the use of hyaluronidase in the treatment of trabectedin extravasation injuries. Although more thorough investigation of adverse effects of trabecetedin extravasation is needed for better comprehension of its damaging impact, the presented data suggest that tacrolimus but mainly DMSO might be suitable antidotes for extravasation injuries caused by trabectedin.

## Data Availability

The original contributions presented in the study are included in the article/supplementary material, further inquiries can be directed to the corresponding author.
